# Strongly Phosphorescent Iridium(III)–Porphyrins – New Oxygen Indicators with Tuneable Photophysical Properties and Functionalities

**DOI:** 10.1002/ejic.201100089

**Published:** 2011-02-23

**Authors:** Klaus Koren, Sergey M Borisov, Robert Saf, Ingo Klimant

**Affiliations:** [a] Institute of Analytical Chemistry and Food Chemistry, Graz University of TechnologyStremayrgasse 9, 8010 Graz, Austria, Fax: +43-316/873-32502 E-mail: sergey.borisov@tugraz.at; [b] Institute for Chemistry and Technology of Materials, Graz University of TechnologyStremayrgasse 9, 8010 Graz, Austria

**Keywords:** Porphyrinoids, Iridium, Phosphorescence, Optical sensors, Oxygen

## Abstract

Synthesis and characterization of four iridium(III)–octaethylporphyrins and a π-extended iridium(III)–benzoporphyrin are presented. Strong room-temperature phosphorescence was observed for all of the complexes with quantum yields of up to 30 %. Axial ligands were introduced to tune the photophysical properties and the solubility. Complexes bearing lipophilic ligands such as pyridine or *N*-(*n*-butyl)imidazole were incorporated into polystyrene to obtain optical oxygen sensors. Covalent coupling of the dye is possible by introduction of ligands with binding domains (1-imidazoleacetic acid). This enabled preparation of a water-soluble oxygen probe (by staining bovine serum albumin) and a trace oxygen sensor (by coupling to amino-modified silica gel).

## Introduction

Strongly luminescent metal complexes are applied as indicators in optical sensors,^1^ as emitters in OLEDs^2^ and as labels.^3^ Consequently, they attract much attention from the scientific community. Among others, metalloporphyrins (especially Pt^II^– and Pd^II^–porphyrins), Ru^II^–polypyridyl complexes^4^ and cyclometallated Ir^III^ compounds^5^ areextensively studied. Phosphorescent complexes based onporphyrins and their derivatives (porphyrin–ketones, porphyrin–lactones, π-extended porphyrins) are very versatile, since different modifications of the porphyrin macrocycle are possible.^6^ Complexes based on cyclometallated Ir^III^ compounds usually possess high luminescence quantum yields but often low absorption coefficients.^7^,^8^

To the best of our knowledge, nothing is known about phosphorescent Ir^III^–porphyrins. This is not surprising, in view of the fact that the synthesis and chemistry of such complexes are more difficult than those of the corresponding Pt^II^– and Pd^II^–metalloporphyrins, which are known to be strongly phosphorescent at room temperature. The question arises whether Ir^III^–porphyrins show any kind of luminescence at room temperature. In fact, the combination of Ir^III^ as central metal and porphyrins as ligands is relatively rare in the literature,^9^–^13^ and mainly the catalytic properties of such complexes have been studied.^14^–^16^ Recently,barely luminescent (quantum yield from 0.03 to 1.2 %) Ir^III^–corrols have been reported.^17^

Besides possessing potentially interesting photophysical properties, complexes combining six-coordinating Ir^III^ and porphyrins also exemplify new synthetic possibilities. In contrast to the square planar Pt^II^– and Pd^II^–porphyrins, two additional ligands are introduced in the Ir^III^–porphyrin system. The axial ligands may influence photophysical properties and affect solubility. Introducing ligands with binding domains or groups suitable for covalent linkage would also be of great interest. In this work, several Ir^III^–porphyrins were synthesized and characterized.

## Results and Discussion

**Scheme 1:**
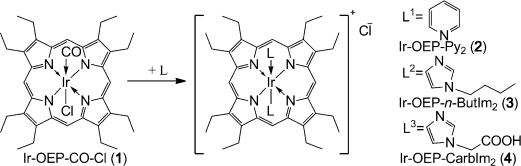
Chemical structures of the four Ir^III^–octaethylporphyrin complexes.

**Figure 1:**
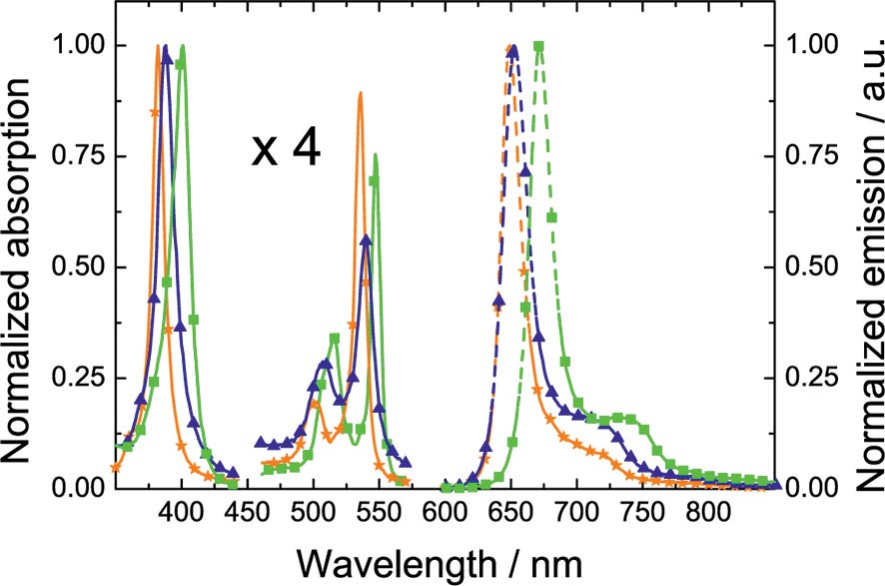
Absorption (solid line) and emission spectra (dashed line) of Ir–OEP–CO–Cl (green squares), Ir–OEP–CarbIm_2_ (blue triangles) and Pt–OEP (orange stars) as reference.

At first, the complexes were studied on the basis of an octaethylporphyrin macrocycle with varying axial ligands (Scheme 1 and Table 1). The carbonyl complex (Ir–OEP–CO–Cl^9^) was employed for preparation of the other complexes by using rather simple and fast ligand exchange reactions. High phosphorescence quantum yields (up to 21 %) were obtained for all the complexes, making them good candidates for oxygen sensing. Photophysical properties were affected by the axial ligands. The positively charged complexes with two identical ligands show quite similar properties in contrast to neutral Ir–OEP–CO–Cl. In fact, despite slightly lower luminescence quantum yield, Ir–OEP–CO–Cl has a significantly longer decay time (*τ*_0_ = 97 μs). Also, the absorption and emission bands are bathochromically shifted (Figure 1 and Table 1). In contrast to those of the well-known Pt^II^–octaethylporphyrin (Pt–OEP), the absorption peaks of all Ir^III^–porphyrins are slightly broader (by about 5 nm at FWHM of the Soret band) and bathochromically shifted. This enables excitation with visible light (400 to 405 nm) via the strongly absorbing Soret band for all Ir^III^–octaethylporphyrins.

**Table 1:** Photophysical properties of Ir^III^–octaethylporphyrins

	*λ*_max_ (abs) /nm (*ε* /10^–3^cm^–1^m^–1^)[b]	*λ*_max_ (em) /nm[a]	*τ*_0_ /μs	*Φ* /%[a]
Ir–OEP–CO–Cl	404 (165), 518 (15), 550 (31)	672	97[a] (108)[e]	14
Ir–OEP–Py_2_	389 (148), 509 (11), 539 (26.6)	655	40[a] (52)[e]	19.5
Ir–OEP–*n*-ButIm_2_	390 (150), 508 (9.7), 541 (15)	655	27[a] (35)[e]	20
Ir–OEP–CarbIm_2_	388 (142), 507 (10), 538 (18)[c]	652[c]	37[c] 26[d]	21[c] 8[d]
Pt–OEP	382 (214), 503 (9.5), 536 (42.5)	649	75[a] (86)[e]	41.5
Pd–OEP	395 (127), 513 (9), 547 (32)	669	(1217)[e]	≈ 19

[a] Diluted solutions of toluene.

[b] CHCl_3_.

[c] EtOH.

[d] Aqueous buffer (pH 7.3) at room temperature.

[e] In polystyrene at 25 °C.

**Figure 2:**
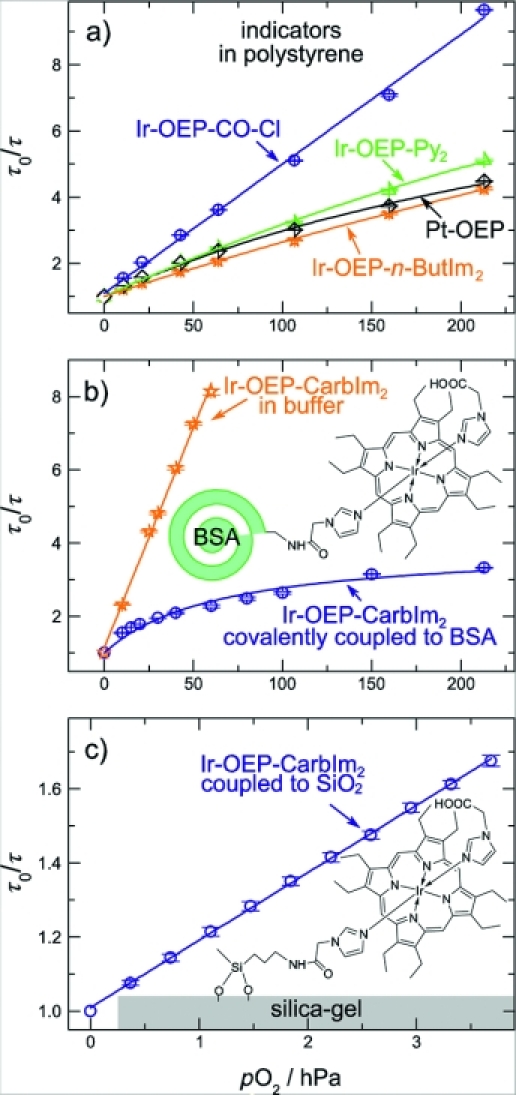
Stern–Volmer plots for the sensing materials based on Ir^III^–porphyrins; Pt–OEP is used as a reference.

Axial ligands can also be used to change the solubility or to introduce binding groups. Similarly to Ir–OEP–CO–Cl, complexes bearing pyridine or *N*-(*n*-butyl)imidazole as axial ligands are well-soluble in organic solvents such as acetone, chloroform and toluene. Therefore, these complexes can be incorporated into polystyrene or other polymers to yield oxygen sensors. Stern–Volmer plots for the oxygen-sensing materials based on the above indicators in polystyrene are shown in Figure 2a. They are compared to the established Pt–OEP in the same matrix.

Quenching behaviour in polymeric films is usually described by the so-called “two site model”,^18^ which suggests that some dye molecules are less quenchable by the analyte than others, leading to nonlinear calibration curves.

It is noticeable that the Stern–Volmer plots for the presented Ir^III^–porphyrins show higher linearity than the Pt^II^ analogue (Figure 2a). Surprisingly, in the case of Ir–OEP–CO–Cl, a linear Stern–Volmer plot (*R*^2^ = 0.997) from 0 to 100 % air saturation was obtained. Interestingly, the size of the axial ligands seems to affect the second-order-rate quenching constant (*k*_q_= *K*_SV_/*τ*_0_). In fact, *k*_q_ increases when the size of the axial ligands increases (*k*_q_ = 361, 500 and 529 [hPa^–1^ s^–1^] for Ir–OEP–CO–Cl, Ir–OEP–Py_2_ and Ir–OEP–*n*-ButIm_2_, respectively). This could possibly be explained by the participation of the axial ligands in the energy transfer reaction.

Axial ligands can also be used to introduce polar groups or binding moieties. For example, an imidazole ligandbearing a carboxyl group renders the porphyrin soluble in polar solvents such as ethanol, methanol and even in aqueous buffer (physiological pH).

The solubility in polar media and the presence of the carboxyl group enable coupling to biomolecules such as proteins, antibodies or lipids. To demonstrate its binding capability, Ir–OEP–CarbIm_2_ was coupled to bovine serum albumin, BSA, (*τ*_0_ = 24 μs). As expected, the quenching efficiency decreases upon binding to BSA, because the dye is better shielded from oxygen (Figure 2b). The highly nonlinear calibration plot can be explained by the variety of the linking positions to the protein, each having different oxygen accessibility. Protein- or peptide-bound oxygen-sensitive dyes are interesting tools to measure, for example, cell respiration,^19^ particularly due to their small size and good solubility in biological media.

Evidently, covalent coupling is not only attractive for biomolecules but also for polymers or functionalized surfaces. Previously, we demonstrated that trace oxygen sensors can be designed by covalently immobilizing Pt^II^– and Pd^II^–porphyrin complexes on the surface of amino-modified silica gel.^20^ For the first time Ir–OEP–CarbIm_2_ enables coupling to silica gel via the axial ligand (*τ*_0_= 26 μs) instead of modifying the porphyrin macrocycle. The obtained sensor is sensitive to small oxygen concentrations (Figure 2c) and features a highly linear calibration plot (*R*^2^ = 0.999).

**Figure 3:**
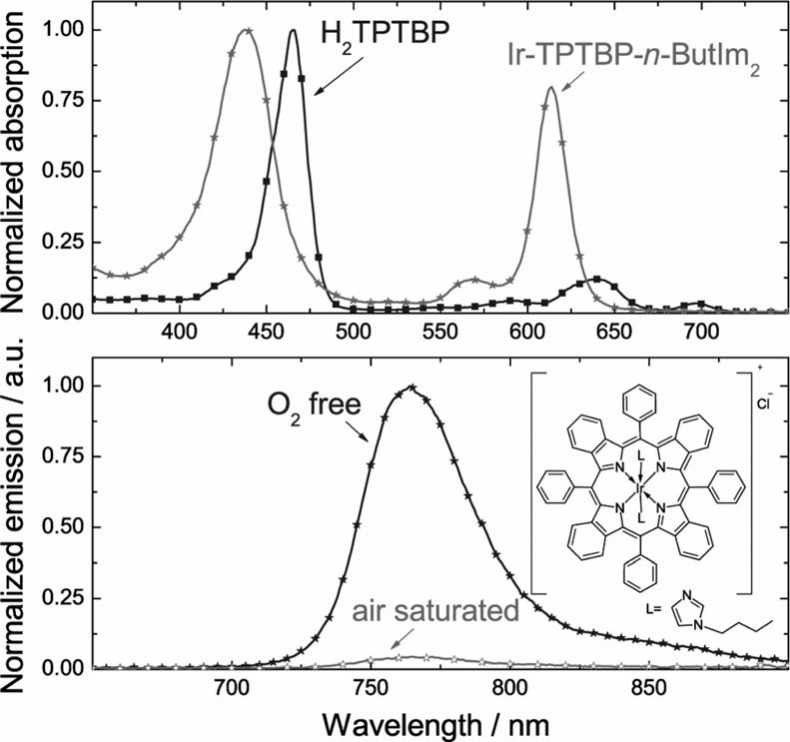
(a) Absorption spectra of the free ligand H_2_TPTBP and Ir–TPTBP–*n*-ButIm_2_; (b) emission spectra of Ir–TPTBP–*n*-ButIm_2_ in toluene solution.

Finally, the combination of Ir^III^ and a π-extended benzoporphyrin was investigated. Pt^II^– and Pd^II^–benzoporphyrins are known to emit in the near infrared (NIR) part of the spectrum.^21^,^22^ NIR-emitting complexes are particularly interesting, as they enable subcutaneous measurements. In this work, we combined Ir^III^ with tetraphenyltetrabenzoporphyrin and chose *N*-(*n*-butyl)imidazole as axial ligand (Ir–TPTBP–*n*-ButIm_2_). Unfortunately, bonding of the axial ligands seems to be weaker in the case of the benzoporphyrin, and they can be partly replaced, for example, by solvent molecules during the purification. Nevertheless, the results presented confirm that Ir^III^ was complexed by the porphyrin (Figure 3). Strong NIR phosphorescence was observed (quantum yield ca. 30 %, *τ*_0_ ca. 23 μs) for Ir–TPTBP–*n*-ButIm_2_. Thus, the Ir^III^–porphyrin complexes represent significantly stronger emitters than the Ir^III^–corrols (quantum yield less than 1.2 %).^17^ Since the positions of the phosphorescence maxima of the Ir^III^–benzoporphyrin and the Ir^III^–corrols are similar, such a huge difference in the quantum yield cannot be explained by the lower energy gap between the triplet excited state and the ground state for the corrols (*λ*_max_ ≈ 790 nm).^17^ The smaller cavity size of the corrols may result in nonplanarity of the Ir^III^ corrols, which may promote nonradiative deactivation.

## Conclusions

It can be concluded that Ir^III^–porphyrin complexes are strong room-temperature emitters. In contrast to the well-known Pt^II^– and Pd^II^–porphyrins, the Ir^III^ complexes bear axial ligands that have pronounced effects on the photophysical properties and solubility of the dyes. They can also be used to introduce functional groups to enable, for example, covalent coupling. The new dyes are particularly promising as indicators for oxygen sensors with tailor-made sensitivity.

## Experimental Section

Ir–OEP–Cl–CO (**1**) was synthesized as described in the literature.^9^ Complexes **2**, **3** and **4** were obtained by ligand exchange reactions. Ligand exchange was accelerated by using a large excess of the ligands. Ir–TPTBP–*n*-ButIm_2_ was synthesized by metallating the free porphyrin (H_2_TPTBP^23^) and subsequent replacement of the axial ligands. Ir–OEP–CarIm_2_ was coupled to amino-modified silica gel particles as well as to BSA through an amide bond by using 1-ethyl-3-[3-(dimethylamino)propyl]carbodiimide and *N*-hydroxysuccinimide as coupling reagents.

**Supporting Information** (see footnote on the first page of this article): Materials and instrumentation, detailed synthetic procedures, characterization of complexes, NMR and HRMS spectra, sensor preparation, measurement setup.
