# Supplementary material for: Strongly Phosphorescent Iridium(III)–Porphyrins – New Oxygen Indicators with Tuneable Photophysical Properties and Functionalities
Source: Eur J Inorg Chem. 2011 Feb 23;2011(10):1531–4. doi: 10.1002/ejic.201100089 (PMC3318677; doi:10.1002/ejic.201100089)
Supplement: Supplementary file 1 [file ejic2011-1531-SD1.pdf]

Eur. J. Inorg. Chem. **2011** · © WILEY-VCH Verlag GmbH & Co. KGaA, 69451 Weinheim, 2011 · ISSN 1434–1948

SUPPORTING INFORMATION

DOI: 10.1002/ejic.201100089
Title: Strongly Phosphorescent Iridium(III)–Porphyrins – New Oxygen Indicators with Tuneable Photophysical Properties and Functionalities
Author(s): Klaus Koren, Sergey M. Borisov,* Robert Saf, Ingo Klimant

Experimental Section ____ *3*

Materials ____ **3**

Methods ____ **3**

Synthesis and Analysis ____ **4**

Synthesis of Chloro-(octaethylporphyrinato)-carbonyliridium(III) (Ir-OEP-Cl-CO) (**1**) ____ 4

Synthesis of bis-pyridino-octaethylporphyrinatoiridium(III) chloride (Ir-OEP-Py_2) (**2**) ____ 5

Synthesis of bis-(N-(n-Butyl)imidazolo)-octaethylporphyrinatoiridium(III) chloride (Ir-OEP-n-$ButIm_2$) (**3**) ____ 7

Synthesis of octaethylporphyrinatoiridium(III) bis-1-Imidazoleacetic acid (Ir-OEP-$CarbIm_2$) (**4**) ____ 10

Synthesis of bis- (N-(n-Butyl)imidazolo)-tetraphenyltetrabenzoporphyrinatoiridium(III) chloride (Ir-TPTBP-n-$ButIm_2$) (**5**) ____ 12

Sensor preparation and coupling reactions ____ **14**

Coupling of Ir-OEP-$CarbIm_2$ to BSA ____ 14

Coupling of Ir-OEP-$CarbIm_2$ to aminomodified silica gel particles ____ 14

Preparation of sensor films ____ 14

References ____ **14**

Experimental Section

Materials

1,5-Cyclooctadien, pyridin, 2-ethoxyethanol and diphenylether were purchased from Aldrich (www.sigmaaldrich.com); N-hydroxysuccidinimide (NHS) from Fluka ; N-(n-butyl)imidazole and iridium trichloride hydrate from ABCR (www.abcr.de); 1-imidazoleacetic acid and 1-ethyl-3(3-dimethylaminopropy) carbodiimide hydrochloride (EDC) from TCI (www.tcieurope.eu); polystyrene (MW. 250000) from Fisher Scientific (www.fishersci.com); poly(ethylene glycol terephthalate) support (Mylar®) from Goodfellow (www.goodfellow.com); platinum(II) octaethylporphyrin (Pt-OEP), palladium(II) octaethylporphyrin (Pd-OEP) and octaethylporphyrine ($OEPH_2$) from Frontier Scientific (www.frontiersci.com); bovine serum albumin (BSA) from Roth (www.carl-roth.de). $[Ir(COD)(\mu\text{-}Cl)]_2$ was synthesized as described in the literature[1]. Silica-gel 60 (0.063-0.200 mm) was purchased from Merck (www.merck.de) and neutral aluminum oxide (50-200 µm) was purchased from Acros Organics (www.acros.com). All other solvents were from Roth (www.carl-roth.de) and used without further purification. Throughout this work deionized water was used.

Methods

Mass spectrometry was performed on a Micromass TofSpec 2E Time-of-Flight Mass Spectrometer. The instrument is equipped with a nitrogen laser (337nm wavelength, operated at a frequency of 5 Hz), and a time lag focusing unit. Ions were generated by irradiation just above the threshold laser power. Positive ion spectra were recorded in reflectron mode applying an accelerating voltage of 20 kV and externally calibrated with a suitable mixture of poly(ethyleneglycol)s (PEG). The spectra of 100–150 shots were averaged to improve the signal-to-noise ratio. Analysis of data was done with MassLynx-Software V3.5 (Micromass/Waters, Manchester, UK). Samples were dissolved in THF ($C = 0.1 mgmL^{-1}$), dithranol or α- Cyanocinnamic acid was used as matrix (C=10mgmL−1 in THF), respectively. The solutions were mixed in the cap of a microtube in the ratio of 1µL:10µL. Then, 0.5µL of the resulting mixture were deposited on the sample plate (stainless steel) and allowed to dry under air. LDI mass spectrometry was performed on the same instrument.
Absorption spectra were measured at a Cary 50 UV–VIS spectrophotometer (www.lzs-concept.com). Emission spectra were acquired on a Hitachi F-7000 fluorescence spectrometer (www.inula.at) equipped with a red-sensitive photo-multiplier R 928 from Hamamatsu (www.hamamatsu.com). The emission spectra were corrected for the sensitivity of the PMT which was calibrated using a halogen lamp. Relative luminescence quantum yields were determined using a solution of Pt-OEP in toluene as a standard (quantum yield = 41.5%). The solutions of the dyes were thoroughly deoxygenated by bubbling nitrogen through.
Luminescence phase shifts for the dyes in solutions were measured with a two-phase lock-in amplifier (SR830, Stanford Research Inc., www.thinksrs.com). Excitation was performed with the light of a 405 nm (for OEP complexes) or a 450 nm (for benzoporphyrin complexes) LED which was sinusoidally modulated at frequencies of 2.5, 5 and 8 kHz. A bifurcated fiber bundle was used to guide the excitation-light to the cuvette and to guide back the luminescence after passing the OG630 (Schott), for OEP complexes, or RG 9 (Schott), for benzoporphyrin complexes, glass filter. The luminescence was detected with a photo-multiplier tube (H5701-02, Hamamatsu, www.sales.hamamatsu.com). Temperature was controlled by a cryostat ThermoHaake DC50. Gas calibration mixtures were obtained using a gas mixing device (MKS, www.mksinst.com). Trace oxygen concentrations were measured as reported elsewhere[2]

Synthesis and Analysis

Synthesis of Chloro-(octaethylporphyrinato)-carbonyliridium(III) (Ir-OEP-Cl-CO) (1)

Ir-OEP-Cl-CO (**1**) was synthesized as described in the literature[3]. In brief, 200 mg of $OEPH_2$ (0.374 mmol) and 300 mg of $[Ir(COD)(\mu\text{-}Cl)]_2$ (0.482 mmol) were refluxed in 200 mL of p-xylene for about 8 hours. Reaction progress was monitored via UV-Vis absorption. After solvent removal silica gel chromatography was performed. Starting material and byproducts were eluted using toluene. The product was eluted with toluene:acetone (95 : 5). yield: 105 mg (35%)

UV-Vis: (toulene), λ/nm (relative intensity): 404 (1.00), 518 (0.09), 550 (0.19); ^{1}H NMR (300 MHz, $CDCl_3$), ppm: 10.31 (s; 4H), 4.15 (q; 16H), 2.02 (t; 24H)

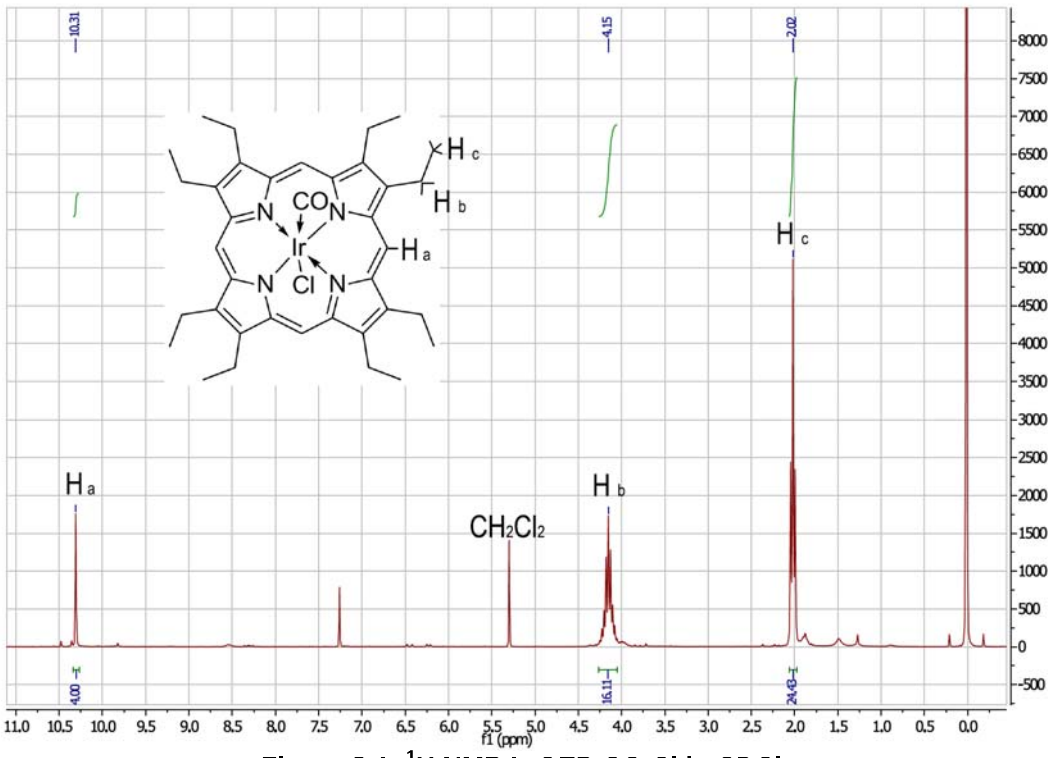

Figure S 1: ^{1}H NMR Ir-OEP-CO-Cl in $CDCl_3$

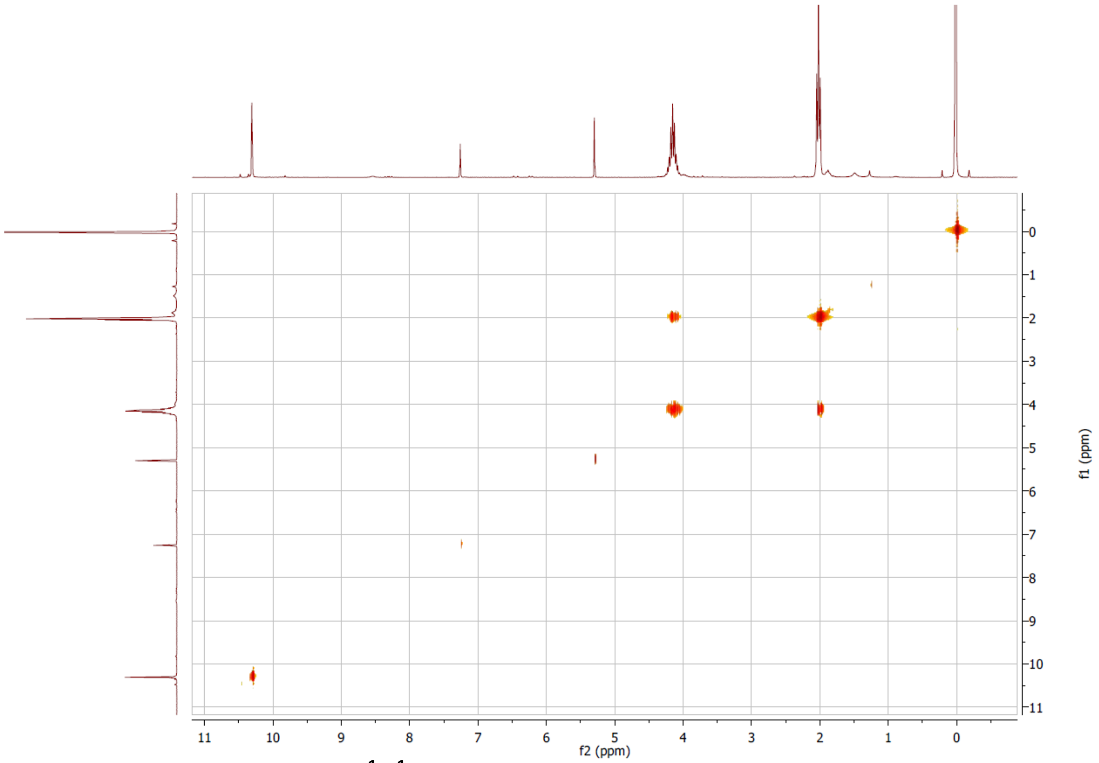

Figure S 2: $^1H^1H$ COSY NMR Ir-OEP-CO-Cl in $CDCl_3$

Synthesis of bis-pyridino-octaethylporphyrinatoiridium(III) chloride (Ir-OEP-Py_2) (2)

47 mg of Ir-OEP-CO-Cl (0,059 mmol) was refluxed in 5 mL of pyridine for 24 hours. The product was precipitated with 50 mL of H_2O and 10 mL of saturated NaCl solution. The precipitate was washed 3 times with water, dried and purified on silica gel. Byproducts were removed using CH_2Cl_2 and CH_2Cl_2:acetone (1:1). The product eluted using acetone:MeOH (95:5). 34 mg (yield: 65%) of Ir-OEP-Py_2 were obtained.

UV-Vis: ($CHCl_3$), λ/nm (relative intensity): 389 (1.00), 509 (0.075), 539 (0.18)

HRMS (MALDI): m/z $[M]^+$ calc. 881.4016 , found 881.4037

^1H NMR (300 MHz, $CDCl_3$), ppm: 10.19 (s; 4 H), 6.03 (t; 2H), 4.94 (t; 4H), 4.10 (q; 16H), 1.91 (t; 24H), 0.23 (d; 4H)

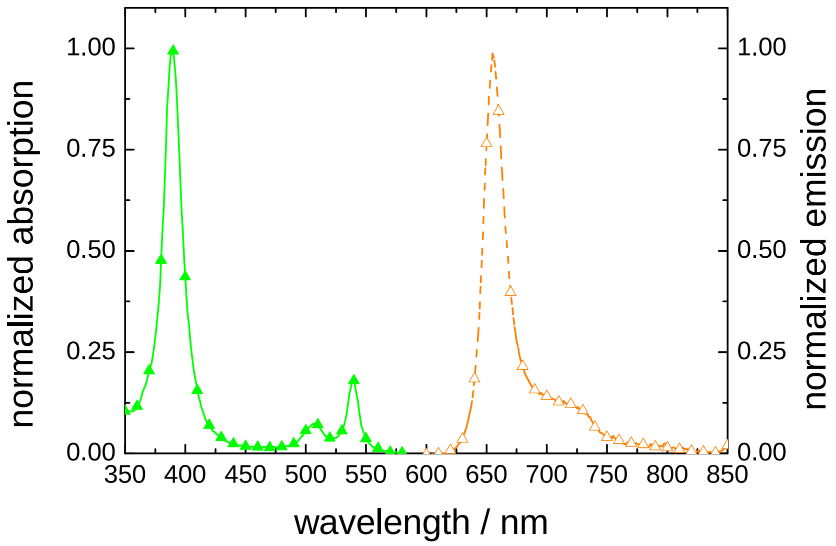

Figure S 3: Normalized absorption (full line) and emission (dashed line) spectra of Ir-OEP-Py$_2$

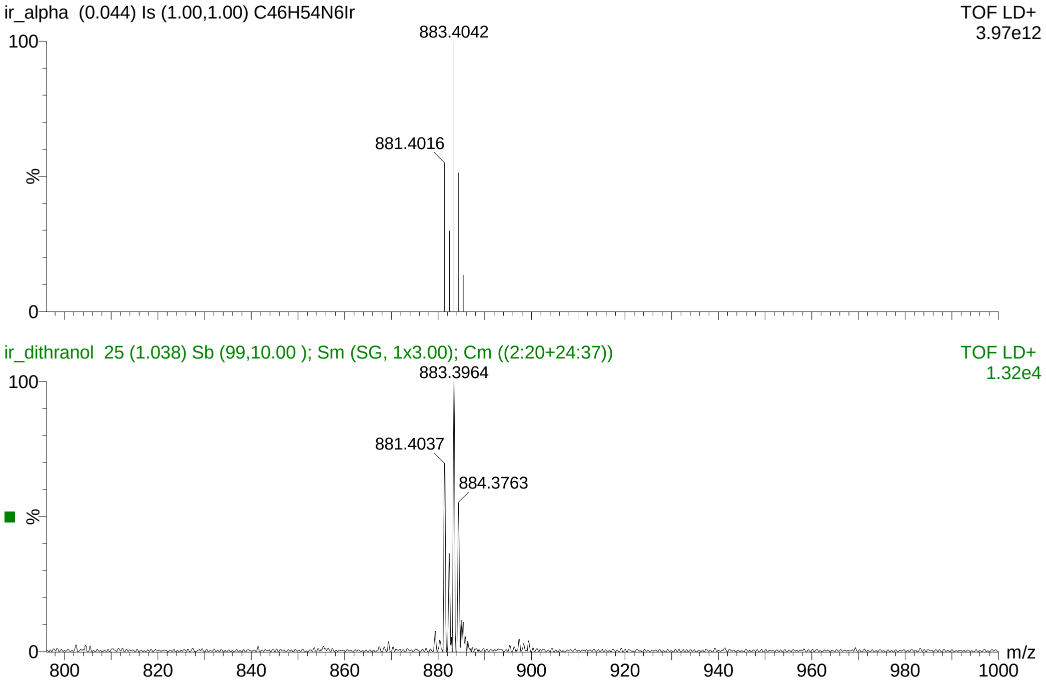

Figure S 4: top: calculated isotope pattern for $C_{46}H_{54}N_6Ir$; bottom: measured isotope pattern for Ir-OEP-Py$_2$

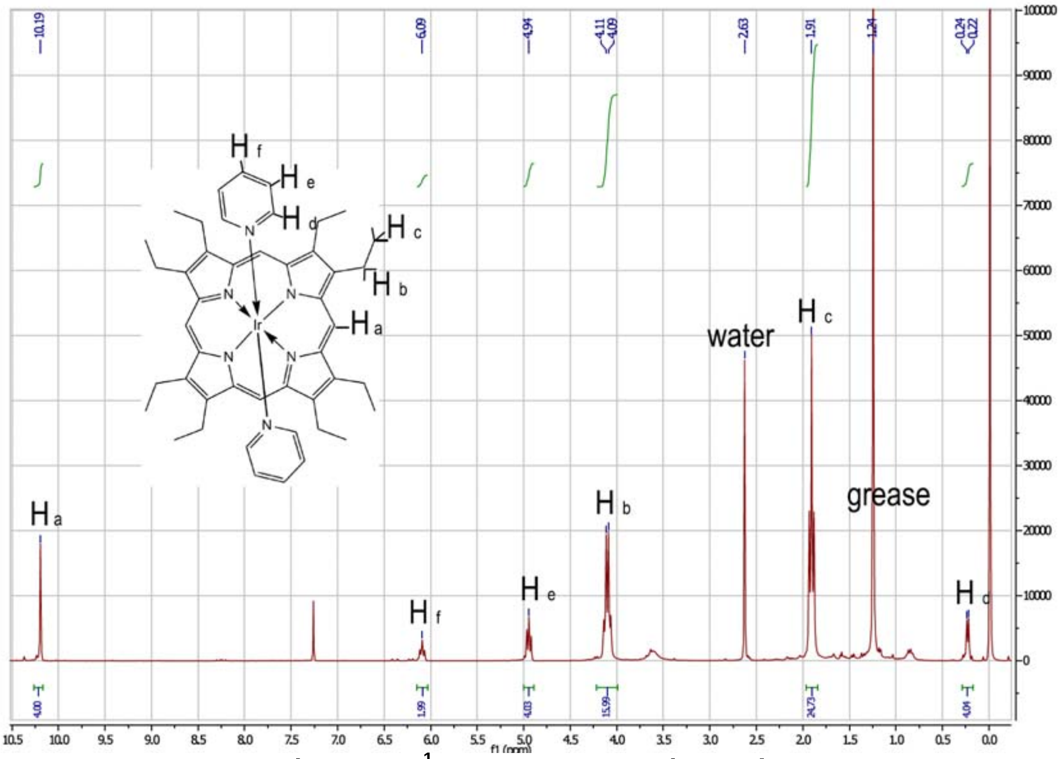

Figure S 5: ^{1}H NMR Ir-OEP-Py$_2$ in $CDCl_3$

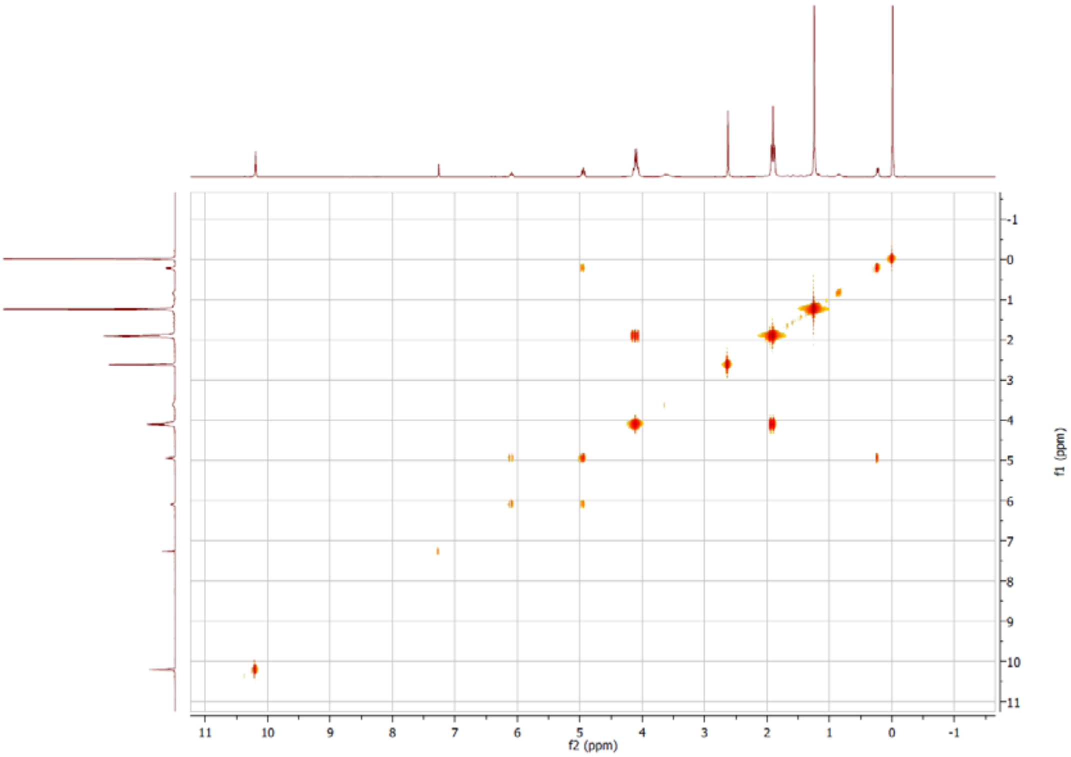

Figure S 6: $^1H^1H$ COSY NMR Ir-OEP-Py$_2$ in CDCl$_3$

Synthesis of bis-(N-(n-Butyl)imidazolo)-octaethylporphyrinatoiridium(III) chloride (Ir-OEP-n-ButIm$_2$) (3)

80 mg of Ir-OEP-CO-Cl (0.10 mmol) was dissolved in 5 mL of N-(*n*-Butyl)imidazole and stirred at 120° C for one hour. The mixture was precipitated with 50 mL of H_2O and 10 mL of saturated NaCl solution. The precipitate was washed 3 times with water, dried and purified on Al_2O_3. Starting material was removed using CH_2Cl_2:acetone (1:1), the product eluted using acetone:MeOH (95:5). 58 mg of Ir-OEP-n-ButIm$_2$ (yield: 60%) were isolated.

UV-Vis: ($CHCl_3$), λ/nm (relative intensity): 390 (1.00), 508 (0.065), 541 (0.10)

HRMS (MALDI): m/z $[M]^+$ calc. 971.5173 , found 971.5223

^{1}H NMR (300 MHz, $CDCl_3$), ppm: 10.08 (s; 4 H), 4.59 (broad s; 2H), 4.08 (q; 16H), 2.22 (t; 4H), 1.90 (t; 24H), 0.45 (broad s; 2H), 0.28 – 0.15 (m; 10H), 0.13 (s; 2H), -0.16 (hex; 4H).

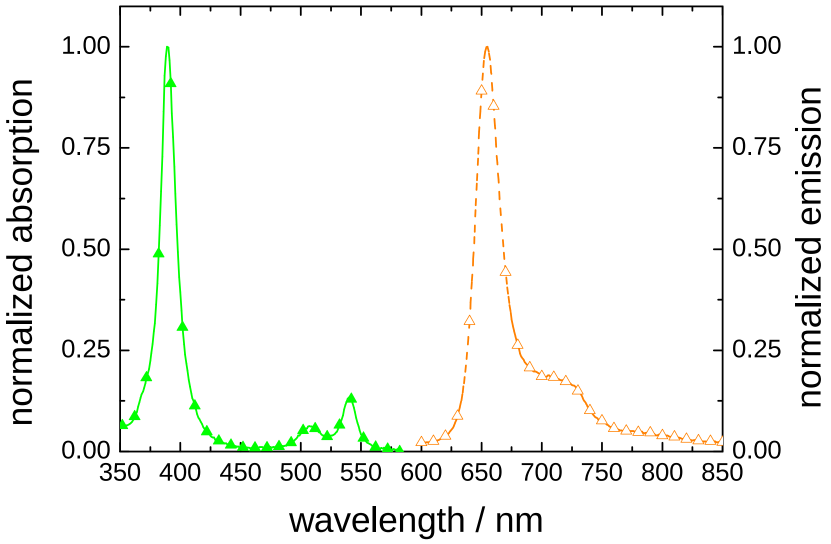

Figure S 7: Normalized absorption (full line) and emission (dashed line) spectra of Ir-OEP-n-ButIm$_2$

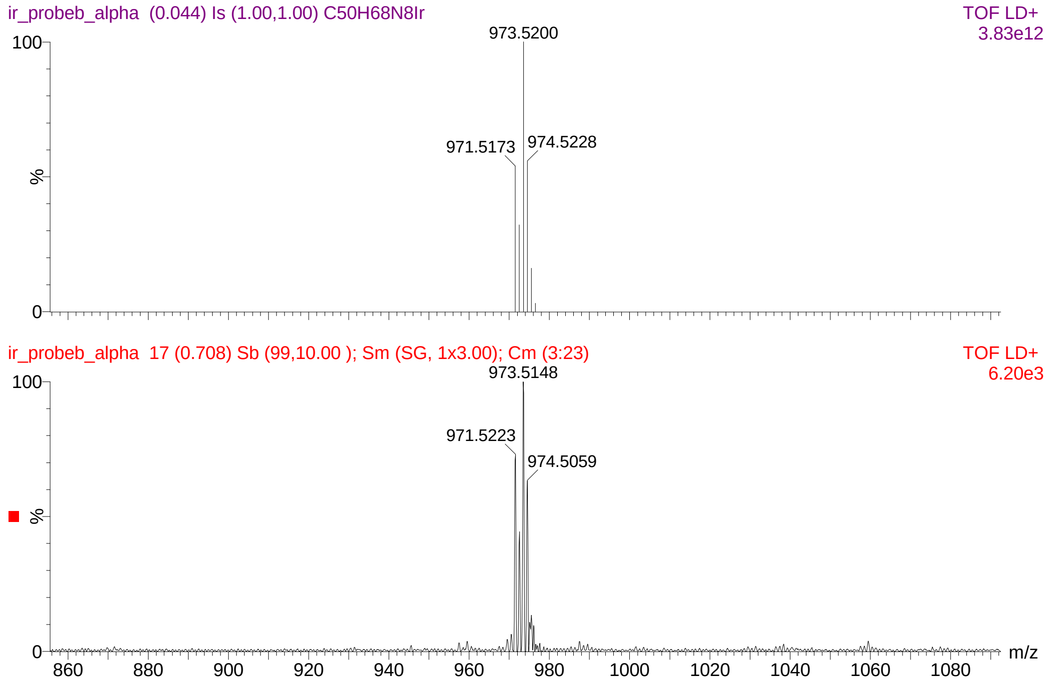

Figure S 8: top: calculated isotope pattern for $C_{50}H_{68}N_8Ir$; bottom: measured isotope pattern for Ir-OEP-n-ButIm$_2$

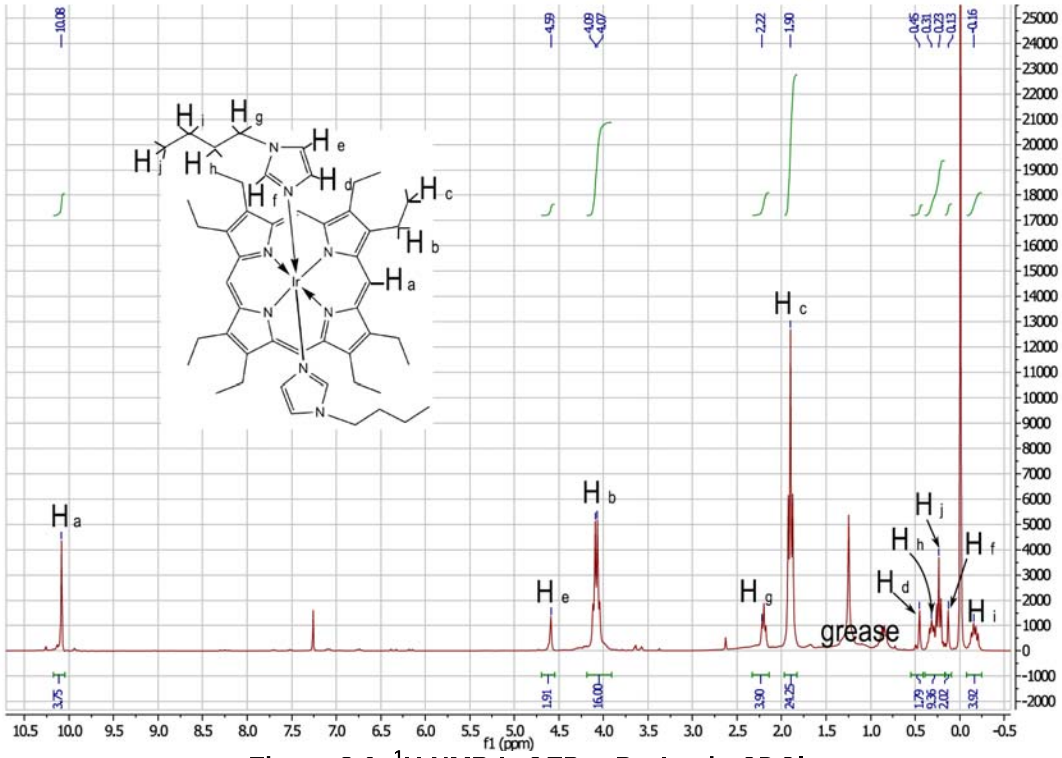

Figure S 9: ^{1}H NMR Ir-OEP-n-ButIm$_2$ in CDCl$_3$

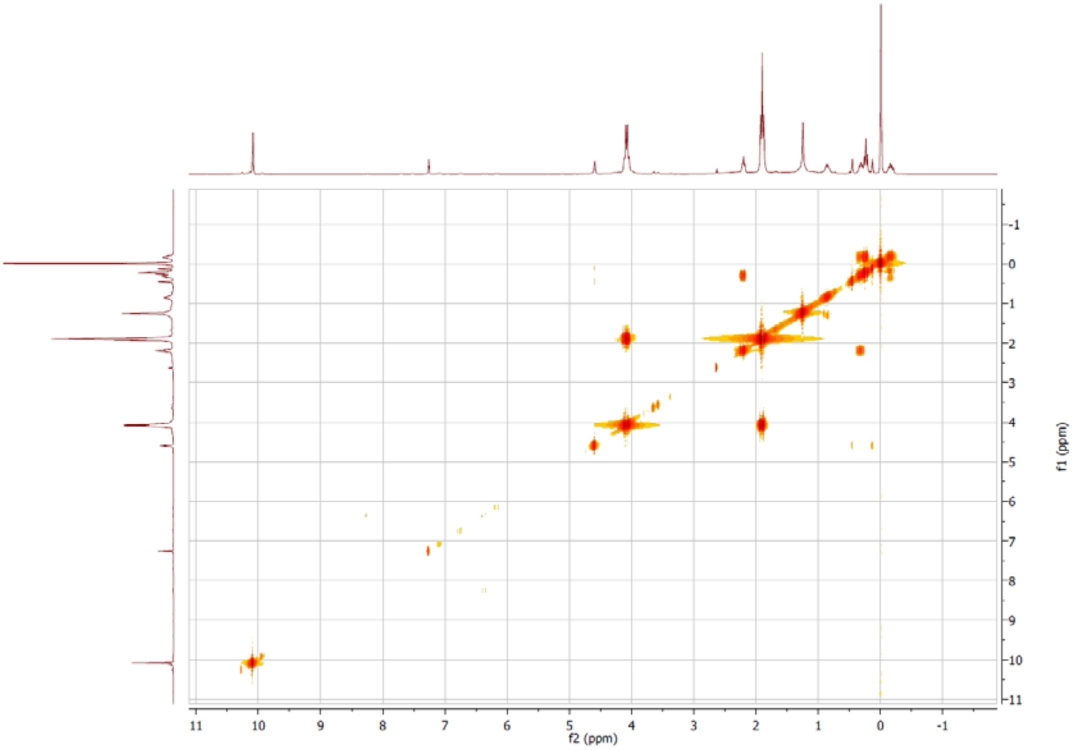

Figure S 10: ^{1}H^{1}H COSY NMR Ir-OEP-n-ButIm$_2$ in CDCl$_3$

Synthesis of octaethylporphyrinatoiridium(III) bis-1-Imidazoleacetic acid (Ir-OEP-CarbIm$_2$) (4)

103 mg of Ir-OEP-CO-Cl (0.138 mmol) and 500 mg of 1-Imidazoleacetic acid (3.96 mmol) were dissolved in 20 mL of 2-ethoxyethanol. The solution was refluxed for 1 hour. After cooling to room temperature the solution was poured into a mixture of H_2O and saturated NaCl solution (1:1, 100 mL in total). After stirring for several minutes the precipitated product was separated via centrifugation and washed three times with dest H_2O and dried. 40 mL of water and 2 mL of 1 M NaOH were added to the obtained solid. After ultrasonification for 30 minutes the soluble and insoluble fraction were separated via centrifugation. The soluble fraction was collected. After adding 4 mL of 1 M HCl the product precipitated and was collected via centrifugation and washed three times. After drying 35 mg (yield: 26%) of Ir-OEP-CarbIm$_2$ were obtained.

UV-Vis: (EtOH), λ/nm (relative intensity): 388 (1.00), 509 (0.07), 540 (0.14)

IR (KBr): 1625 cm^{-1} (C=O carboxylic group)

HRMS (MALDI): m/z $[M]^+$ calc. 975.4031 , found 975.4072

^{1}H NMR (300 MHz, d$_4$-MeOD), ppm: 10.22 (s; 4 H), 4.65 (broad s; 2H), 4.13 (q; 16H), 2.90 (s; 4H), 1.94 (t; 24H), 0.11 (broad s; 2H), 0.01 (s; 2H).

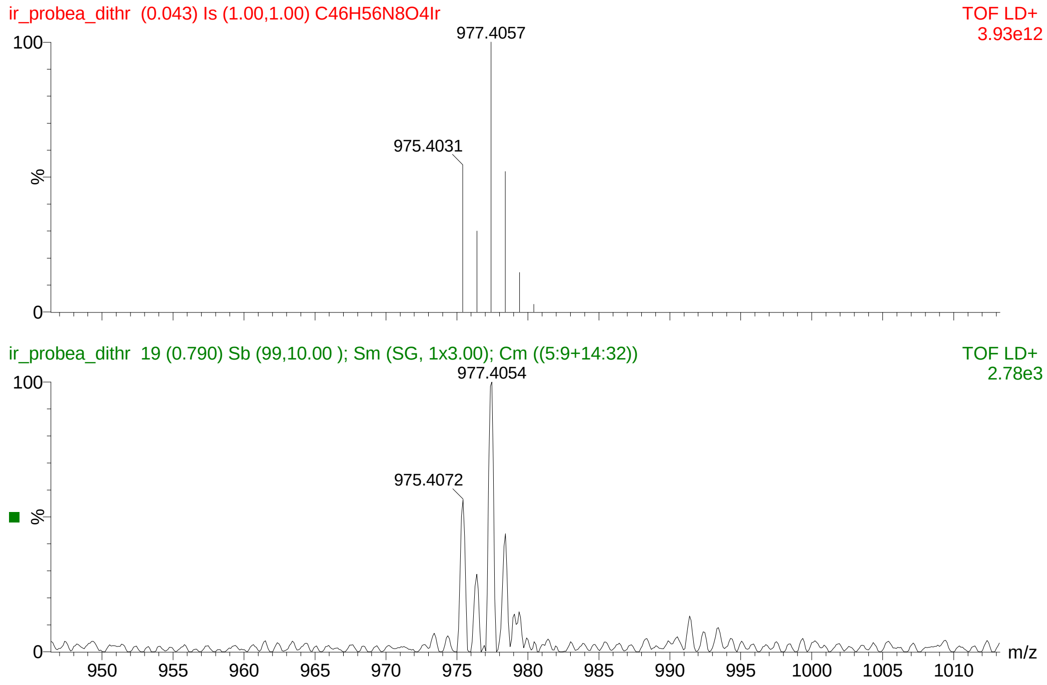

Figure S 11: top: calculated isotope pattern for $C_{45}H_{56}N_8O_4Ir$; bottom: measured isotope pattern for Ir-OEP-CarbIm$_2$

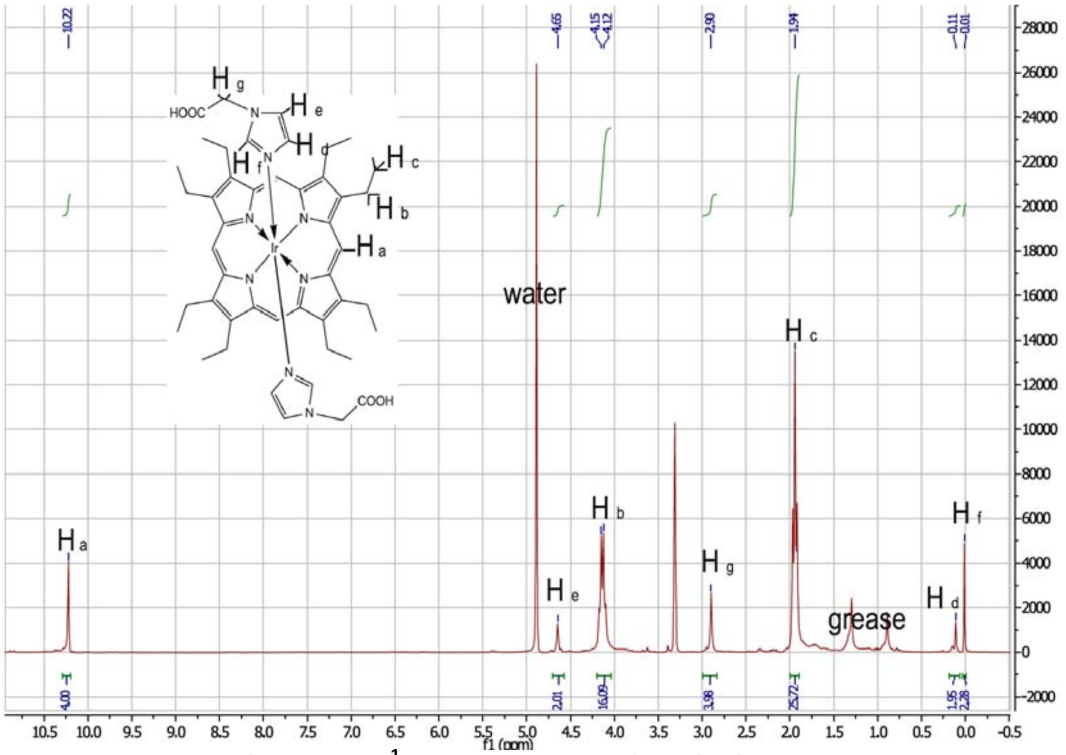

Figure S 12: ^{1}H NMR Ir-OEP-CarbIm$_2$ in d$_4$-MeOD

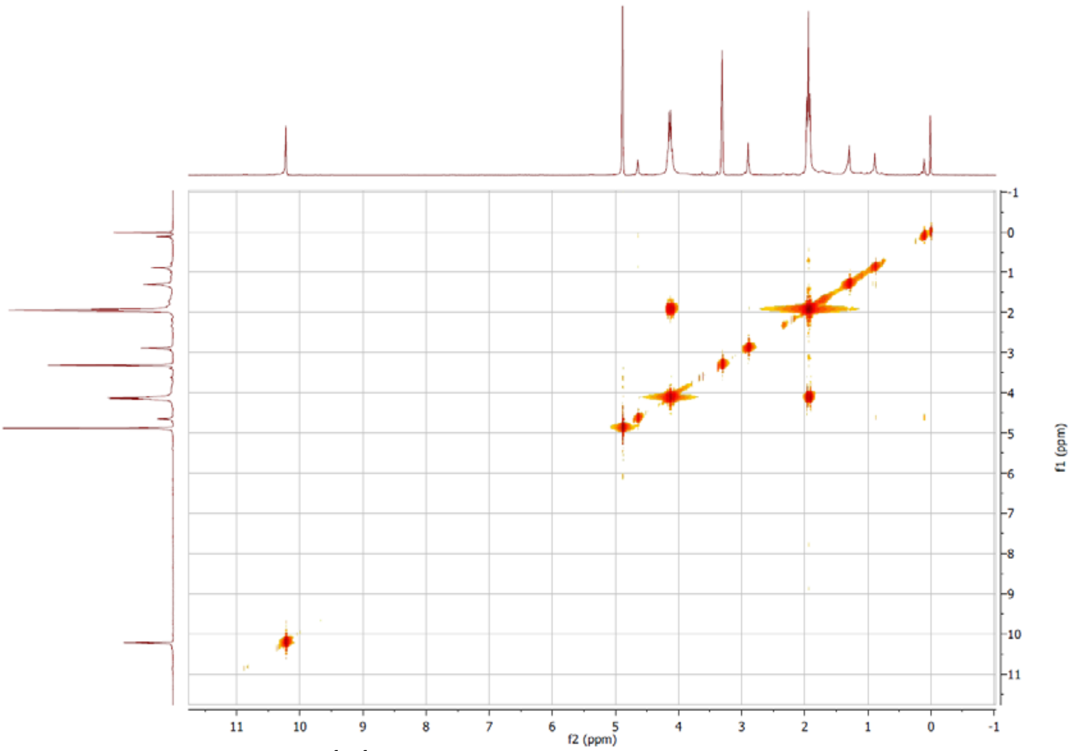

Figure S 13: ^{1}H^{1}H COSY NMR Ir-OEP-CarbIm$_2$ in d$_4$-MeOD

Synthesis of bis- (N-(n-Butyl)imidazolo)-tetraphenyltetrabenzoporphyrinatoiridium(III) chloride (Ir-TPTBP-n-ButIm$_2$) (5)

Tetraphenyltetrabenzoporphyrin (H_2TPTBP) was synthesized as reported previously[4]. Side reactions in the reported synthesis lead to benzyl adducts (C_7H_6 groups are fused to the porphyrin) that contaminate the porphyrin, but do not influence the photophysical properties of the ligand.

100 mg of H_2TPTBP (0.123 mmol) and 125 mg of [Ir(COD)(μ-Cl)]$_2$ (0.186 mmol) were dissolved in 80 mL of ethylene glycol and stirred at 170 °C for 6 hours. Reaction progress was monitored via UV-Vis absorption. The solution was precipitated using 100 mL of water. The precipitate was separated via centrifugation and washed twice with water. After drying the crude product was used for the subsequent step without further purification.

5 mL of N-(n-Butyl)imidazole were added to the crude product. The solution was stirred at 50 °C for 30 minutes. After precipitation with water and subsequent washing the dried product was purified by column chromatography on Al_2O_3 (removal of starting material, eluent: toluene-acetone (1:1); elution of metallated porphyrins, eluent: acetone-MeOH (95:5). After reprecipitation from CH_2Cl_2 with hexane 30 mg of Ir-TPTBP-n-ButIm$_2$ (5) and byproducts (missing one or two of the axial ligands) were obtained.

^{1}H NMR (300 MHz, CDCl3), ppm: 8.31 (d; 8.1H), 8.2 (d; 8.3H), 7.87 (m; 12.5H), 7.13 (m; 9.1H), 7.0 (m; 8H) 4.72 (broad s; 1H), 3.86 (s; 0.3H benzyl adducts), 3.65 (s; 1.03H) 2.30 (t; 1.84H), 0.35 – 0.18 (m; 7.8H), -0.11 (m; 1.98H), -1.39 (broad s; 1.2H).

UV-Vis: ($CHCl_3$), λ/nm (relative intensity): 438 (1.00), 568 (0.12), 614 (0.8)

HRMS (LDI): m/z [M]+ calc. 1253.458 , found 1253.448

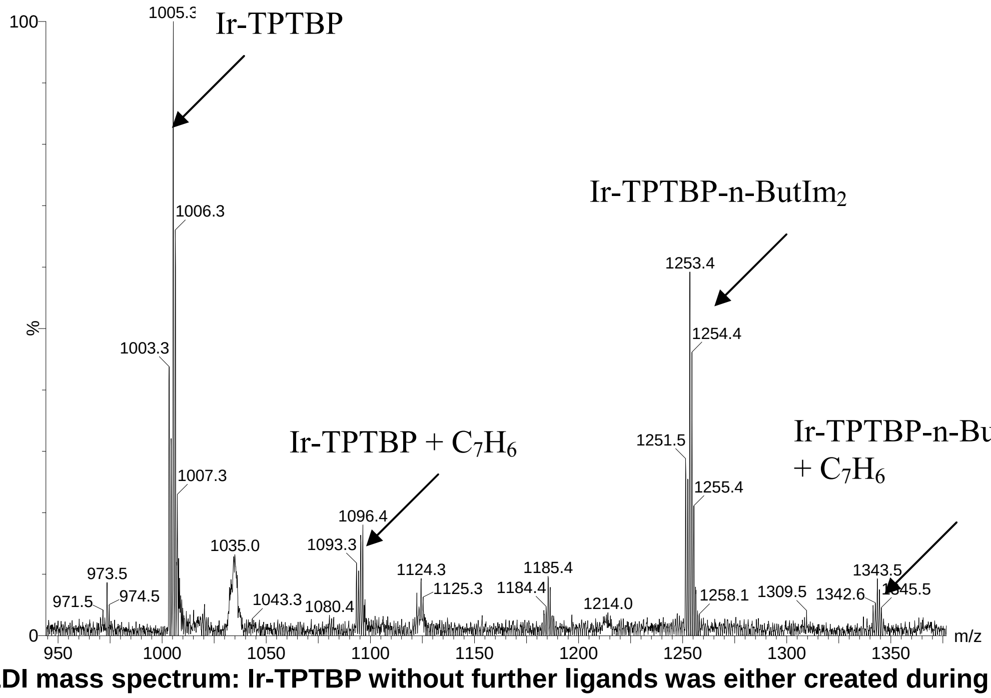

Figure S 14: LDI mass spectrum: Ir-TPTBP without further ligands was either created during the ionization but may also be present initially. The additional C_7H_6 is due to side reactions during the porphyrin synthesis [4]. The Ir(III)-porphyrin with both of the introduced ligands (Ir-TPTBP-n-ButIm$_2$) was also found.

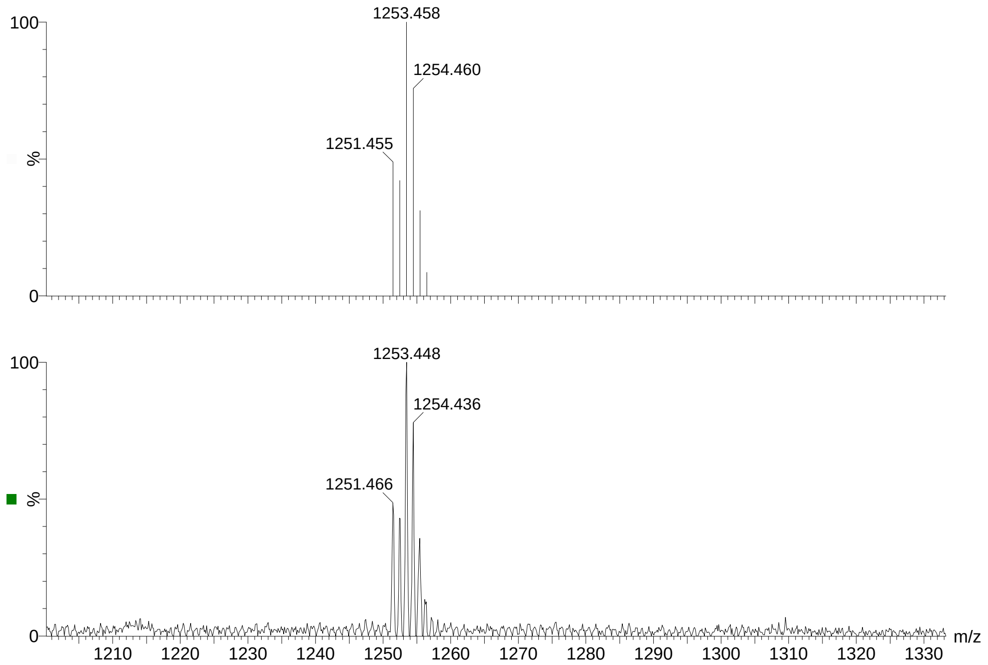

Figure S 15: top: calculated isotope pattern for $C_{74}H_{60}N_8Ir$; bottom: measured isotope pattern for Ir-TPTBP-n-ButIm$_2$

Sensor preparation and coupling reactions

Coupling of Ir-OEP-CarbIm$_2$ to BSA

3.8 mg of EDC (dissolved in 250 µL of phosphate buffer pH 7.3 200 mM), 6 mg of NHS (dissolved in 250 µL of phosphate buffer pH 7.3 200 mM) and 4 mg of Ir-OEP-CarbIm$_2$ (dissolved in 1 mL of EtOH) were combined and mixed at room temperature for 15 minutes. Afterwards 12.4 mg of Bovine Serum Albumin (BSA) (dissolved in 1 mL of phosphate buffer pH 7.3 200 mM) were added. The reaction was carried out at room temperate under continuous stirring for 18 hours.
Protein was purified via size exclusion chromatography with an Amersham pharmacia biotech ÄKTA purifier 900.

Coupling of Ir-OEP-CarbIm$_2$ to aminomodified silica gel particles

Aminomodified silica gel particles (ASP) were obtained as described in the literature[2]. 24 mg ASP (dispersed in 1 mL of phosphate buffer pH 7.3 200mM), 6 mg of EDC (dissolved in 1 mL of phosphate buffer pH 7.3 200 mM), 8 mg of NHS (dissolved in 1 mL of phosphate buffer pH 7.3 200mM) and 2 mg of Ir-OEP-CarbIm$_2$ (dissolved in 1 mL of EtOH) were combined and mixed for 4 hours at room temperature. As a control reaction (blank) the same reaction was carried out without EDC and NHS.
In both cases the particles were separated via centrifugation and washed three times with EtOH and three times with H_2O. While the coupling reaction yielded red colored particles, the control reaction produced virtually uncolored particles.

Preparation of sensor films

The „cocktails“ for coating were prepared by dissolving 1 mg of an indicator and 200 mg of polystyrene in 1800 mg of $CHCl_3$. The cocktails were knife-coated on Mylar support to give, after solvent evaporation, phosphorescent sensor films of ~2.5 µm thickness.
Sensor film containing indicator (4) coupled to silica gel was prepared as followed. 20 mg of the particles were dispersed in 50 mg silicon E4 (www.wacker.com) and 150 mg hexane. The mixture was knife-coated on a glass slide and dried overnight. Finally the sensor film was dried in a vacuum drying chamber for 45 minutes.

References

[1] R. Walter, S. Kirchner, R. Franz, U.S. Patent 6,399,804, 2002

[2] S. M. Borisov, P. Lehner, I. Klimant, Anal. Chim. Acta submitted

[3] H. Ogoshi, J. Setsune, Z. Yoshida, Journal of Organometallic Chemistry 1978, 159, 317-328.

[4] S. M. Borisov, I. Klimant, Dyes and Pigments 2009, 83, 312-316.
